# Agile Methodologies Applied to the Development of Internet of Things (IoT)-Based Systems: A Review

**DOI:** 10.3390/s23020790

**Published:** 2023-01-10

**Authors:** Gleiston Guerrero-Ulloa, Carlos Rodríguez-Domínguez, Miguel J. Hornos

**Affiliations:** 1 Faculty of Engineering Science, State Technical University of Quevedo, Quevedo 120301, Ecuador; 2 Software Engineering Department, Higher Technical School of Computer and Telecommunications Engineering, Aynadamar Campus, University of Granada, 18071 Granada, Spain

**Keywords:** Internet of Things (IoT), development methodologies, agile methodologies, software engineering, Model-Based Engineering, Model-Driven Engineering

## Abstract

Throughout the evolution of software systems, empirical methodologies have been used in their development process, even in the Internet of Things (IoT) paradigm, to develop IoT-based systems (IoTS). In this paper, we review the fundamentals included in the manifesto for agile software development, especially in the Scrum methodology, to determine its use and role in IoTS development. Initially, 4303 documents were retrieved, a number that was reduced to 186 after applying automatic filters and by the relevance of their titles. After analysing their contents, only 60 documents were considered. Of these, 38 documents present the development of an IoTS using some methodology, 8 present methodologies focused on the construction of IoTS software, and 14 present methodologies close to the systems life cycle (SLC). Finally, only one methodology can be considered SLC-compliant. Out of 38 papers presenting the development of some IoTS following a methodology for traditional information systems (ISs), 42.1% have used Scrum as the only methodology, while 10.5% have used Scrum combined with other methodologies, such as eXtreme Programming (XP), Kanban and Rapid Prototyping. In the analysis presented herein, the existing methodologies for developing IoTSs have been grouped according to the different approaches on which they are based, such as agile, modelling, and service oriented. This study also analyses whether the different proposals consider the standard stages of the development process or not: planning and requirements gathering, solution analysis, solution design, solution coding and unit testing (construction), integration and testing (implementation), and operation and maintenance. In addition, we include a review of the automated frameworks, platforms, and tools used in the methodologies analysed to improve the development of IoTSs and the design of their underlying architectures. To conclude, the main contribution of this work is a review for IoTS researchers and developers regarding existing methodologies, frameworks, platforms, tools, and guidelines for the development of IoTSs, with a deep analysis framed within international standards dictated for this purpose.

## 1. Introduction

Several methodologies have been proposed to develop traditional information systems (ISs), some of them being universally known. The first methodology for the development of ISs was the waterfall methodology, presented by Winston Royce [1]. Then, other well-known and used methodologies emerged, such as Spiral [2], Rapid Prototyping (RP) [3,4], and agile methodologies [5,6]. Among the latter, some popular ones are Scrum [7,8,9] and Extreme Programming (XP) [9,10,11]. These methodologies have been widely used for the development of ISs, but with the emergence of Web(-based) Information Systems (WISs), the need for new methodologies also arose. Some examples of such methodologies are the Object-Oriented Hypermedia Design Method (OOHDM) [12,13], Hypermedia Data Bases (HDM) [14,15], Enhanced Object-Relationship Model (EORM) [16,17,18], and Relationship Management Methodology (RMM) [19,20].

Nowadays, a new type of systems is currently emerging, due to the advancement and popularisation of technologies related to the Internet of Things (IoT). In this paper, we name those systems as IoT-based Systems (IoTSs). IoTSs are intended to monitor and control the environment through the deployment of sensors and actuators that can interact with each other and with Internet services. IoTSs allow us to remotely monitor the current state of any physical object or “thing”, modify the conditions of the environment, obtain data to predict or infer events and make decisions in real time [21,22,23,24]. To achieve those goals, physical objects become digital objects that can be manipulated from anywhere and connected to the Internet [22,25,26,27,28]. IoTSs have been applied to multiple fields, such us improving people’s lifestyles, health, work productivity, entertainment, etc. [22,24,25,29,30,31,32,33].

IoTSs are systems that are changing the world and will change it even more in the future. However, to fully exploit the potential of IoT, well-defined development methodologies are required, so as to improve the success rate of the development process and the quality of the resulting system.

Faced with this recent paradigm of systems, the Software Engineering (SE) research field has been tasked to propose methodologies that can specifically cover the development lifecycle of IoTS, since such systems have notable differences with traditional ISs and WISs. It should be noted that IoTSs are composed not only of software applications but have two additional components: hardware (sensors/actuators), and communication mechanisms to allow the interaction between that hardware and the Internet [34,35]. Moreover, the interaction between things and people through well-defined interfaces should also be considered too [29,36,37,38]. In short, there is a need to formulate and validate methodologies for the development of IoTSs.

Therefore, scientists and developers of this new type of systems, namely IoTSs, need a methodology to ensure the quality of their work. At the time of writing, there is no universally adopted methodology for the development of IoTSs. This is evidenced by the many IoTS development methodologies presented in research papers found in ScDBs (see Section 3), and by previous state-of-the-art review papers on IoTS development methodologies [39,40]. Furthermore, among the methodologies found in the literature, only one of them complies with international standards on system and/or software development lifecycles (Section 3 and Section 4).

The following subsections present a background on software engineering, the objectives that guided the writing of this paper, and a state-of-the-art review of previously published works on IoTS development methodologies.

### 1.1. Background

As it was previously mentioned, the first software development methodology was the waterfall methodology, presented by Winston Royce [1]. This methodology is commonly used for the elaboration, manufacture, or construction of any physical product, with the particularity that the original waterfall methodology considers that the developer could return to any previous stages when necessary, even if it could be difficult or unfeasible in many cases [41]. In contrast, hardware construction methodologies do not consider that possibility, since it would involve a great economic impact.

Given the delays and limited achievements in the implementation of the waterfall methodology, it is necessary to identify the reasons for its low impact on the software industry. One of those reasons is that the waterfall methodology encompasses the development of large information systems as a whole [5,42,43,44]. In contrast, others, such as the spiral methodology, help to detect when it is not possible to develop the planned system and, consequently, abandon its development before investing resources in it [10,45].

Another reason is that in the analysis stage of a software system, usually end users (customers) do not have a very clear picture of the functionalities and quality properties that need to be covered. Therefore, the prototyping methodology [46] arose to formalize the presentation of iterative versions of the product to the end users for their evaluation. Prototyping is currently used as part of other methodologies, especially agile methodologies.

Agile methodologies emerged to reduce the risk of not completing the development of large systems due to budget, technological or resource constraints, among other reasons [47,48]. The emergence of agile methodologies made it possible to improve the success rate of software development projects and reduce the budget consumption of projects that are finally abandoned [49,50].

Agile methodologies divide the overall system into deliverables (modules or subsystems), so that the customer uses or checks those deliverables before the entire system is completed. Agile methodologies are based on 4 values and 12 principles included in the Manifesto for Agile Software Development [42]. These methodologies focus only on the development of a part of the overall system, deploying fully functional products for that part of the system in a short period of time (maximum of 4 to 6 weeks).

In addition, some of the characteristics of agile software development methodologies, in contrast with traditional methodologies, are: teams must be small, the deliverables to be developed must be negotiated with the client and changes can be introduced in the project at any time [43,44]. However, in some cases, those features could turn into disadvantages [51]. For example, in an enterprise software development project, a small team of developers, as suggested by agile methodologies (in Scrum, 8 developers), is not sufficient when time is pressing [52]. Additionally, whenever an unexpected change needs to be performed to software, there could be budget issues, since the development team could have to deal with it using the original budget [53]. Moreover, if the decision for the development priority of each deliverable primarily relies on the customer [52,54], this may not help the development team to be productive.

Regarding IoTSs, we found in the analysis presented herein that the authors have usually completed their developments using ad hoc methodologies or without any explicitly mentioned methodology at all. One example is the work of Gea et al. [55], which presents a system developed to integrate existing sensor networks and an intelligent front-end application built with the technology of the moment, but without mentioning the development methodology used. Another example is the work done by Yelamarthi et al. [56] which describes several IoTSs developed for different purposes, including healthcare, structural health monitoring, agriculture, and tourist guidance. However, the research process suggests that no definite methodology has been followed in the development process. Additionally, some authors have developed IoTSs following methodologies not specifically designed for this type of system. A couple of examples of this are the combination of Scrum with XP [57,58] and the combination of Scrum with RP [59,60].

### 1.2. Objectives

This paper aims to guide researchers and developers on methodologies that

- Have been proposed for IoTS development.
- Comply with the life cycle of software systems according to standards issued jointly by the International Organization for Standardisation (ISO), the International Electrotechnical Commission (IEC), and the Institute of Electrical and Electronics Engineers (IEEE). The application of ISO/IEC/IEEE 15289:2019 [61] to the development of IoTSs contributes to the delivery of a quality product on time and within budget [62].
- Consider the specific developmental aspects of IoTSs.

Therefore, we present an in-depth analysis of the state-of-the-art IoTS development methodologies, so that researchers and developers could choose the most appropriate methodology for the development of their IoTSs. Moreover, researchers could work on this analysis to define a methodology specifically designed for the development of IoTSs, and that complies with the ISO/IEC/IEEE 15289:2019 standards and that covers all aspects of the life cycle of such systems [61].

### 1.3. State of the Art in Methodologies to Develop IoTSs

SE is responsible for providing an adequate methodology for the development of all types of computer systems [63,64]. Therefore, researchers in this field have been working to provide optimal methodologies to develop the different types of systems that have recently emerged [64].

In the literature, there are several methodologies for the development of IoTSs. For example, INTER-METH, which was presented by Fortino et al. [65], is a methodology for the development of IoTSs based on the waterfall methodology, making it iterative. Likewise, Test-Driven Development Methodology for IoTSs (TDDM4IoTS), presented by Guerrero-Ulloa et al. [37], is based on the manifesto for agile software development and complies with the stages of the system development lifecycle [66,67]. However, none of them is universally accepted [39,40]. This fact is evident in the state-of-the-art reviews of methodologies for IoTS development found in the ScDBs considered, which are presented below. However, those works pursue different objectives from those set out in this article or follow substantially different methodologies for their study.

Bouanaka et al. [40] present a state-of-the-art review of IoTS development methodologies applied to smart traffic lights. They consider that there is a limited number of methodologies that can be used to develop these types of IoTSs and clearly represent their specific characteristics. The authors provide information to support the decision-making of a small group of developers when deciding on which methodology to follow.

Fortino et al. [39] have also addressed the analysis of existing methodologies for the development of IoTSs. However, the products analysed in the study are based on third-party surveys, rather than on scientific publications. Some aspects that are analysed in the revised methodologies match with those of the present study, such as the development life cycle stages. To unify the terminology found (methodology, framework, platform, tool) during their review process, Fortino et al. [39] refer to ISO/IEC/IEEE 24765 [68], SEBoK (Systems Engineering Body of Knowledge) [69], and PMBoK (Project Management Body of Knowledge) [70,71]. However, none of the previous state-of-the-art review works mention the ISO/IEC/IEEE standards on which they base the analysis.

The present document is proposed as a result of not having found in the literature an exhaustive review of the state-of-the-art IoTSs concluding whether there is a universally adopted methodology for developing IoTSs. This work also aims to present a review of the minimum conditions of exclusion and a thorough analysis of the existing literature. Moreover, this work analysis if the existing proposals adhere to international standards, so as to guarantee the quality of the IoTSs developed.

So far, no IoTS development methodology has been set in ISO/IEC/IEEE 15289:2019 [61] as a standard that unifies the processes specified in ISO/IEC/IEEE 12207:2017 [72] and ISO/IEC/IEEE 15288:2015 [67]. Although none of the proposed methodologies covers all the technical aspects of the software systems life cycle set out in ISO/IEC/IEEE 15289:2019, findings from extant literature suggest that TDDM4IoTS [37] is the only methodology whose IoTS development life cycle follows that standard.

The remainder of this document is organised as follows: Section 2 presents IoTSs developed with traditional software development methodologies. Section 3 discusses existing methodologies for developing IoTSs. Section 4 provides a study of the automated tools and frameworks used for the development of IoTSs. Section 5 is dedicated to architectures for IoTSs, but from a dual point of view: those incorporated into the developed IoTSs and those used in the development methodologies. Finally, Section 6 presents the main conclusions of the analysis carried out.

## 2. Methodologies Designed for the Development of IoTSs

A system development methodology can be defined as “a series of stages of a software or hardware creation following a pattern based on experience and theory of program design” [73]. On the other hand, a possible definition of software development methodology would be “a process of dividing software development work into smaller, parallel, or sequential steps or sub-processes to improve design, product management” [74]. Therefore, both systems and software development methodology may include the predefinition of specific deliverables and artifacts that a project team creates and completes to develop or maintain an application or system [75].

Developing IoTSs using methodologies that are designed for the development of traditional ISs has the great disadvantage that they do not cover specific aspects of IoTSs, such as the design and deployment of hardware (e.g., sensors, actuators, processors, and so on) in the environment to be controlled. Moreover, other aspects that, although not unique to IoTSs, are essential in this type of system, such as the incorporation of artificial intelligence (AI) techniques to help the system in decision-making and to be context-aware, i.e., it reacts appropriately according to the context or the existing conditions in the environment [39,42]. Therefore, and if we also consider the heterogeneity of the components and application domains of IoTSs [76,77], it is obvious to conclude that professionals from different areas should be part of their development team in order to carry out the activities included in the different stages of its development, either to act throughout the whole life cycle or to carry out specific tasks [78].

### 2.1. Stages or Processes of the Software System Development Life Cycle

To make an analysis of system development methodologies, we could go back to the 40s, when the digital or general-purpose computer called EDVAC was created [79], or to the 50s and 60s, when many programming languages appeared, such as Fortran, Cobol or Basic, to name a few [80]. The first software development methodologies for desktop systems were defined according to the paradigm on which the programming language to implement the system was based. For example, to develop using structured programming languages, structured development methodologies appeared [37,81], while for development using object-oriented programming languages, object-oriented methodologies appeared [37,82]. Subsequently, the methodologies were reoriented according to the type of system to be developed, such as desktop or web [37].

The *waterfall* methodology comprised the following phases or stages: (a) system requirements analysis, (b) software requirements analysis, (c) preliminary program design, (d) system analysis, (e) software design, (f) coding, (g) testing, and (h) operation. 

Subsequently, agile methodologies, among which some popular ones are XP, Kanban, and the Scrum framework [83], consider that the values and principles of the agile manifesto should be present in the development methodology of any software product that can be broken down and developed into parts. In the development of any IoTS, it is necessary to consider: the deployment of different hardware components (e.g., sensors, actuators, processors, and so on) in the environment to be controlled, communication and interaction between objects or “ things” connected, and the development of the means for user interaction with the system (e.g., web application, mobile application, and so on) [22,84,85,86], among other aspects, which will eventually become deliverables. These deliverables will be developed by carrying out different activities. Therefore, we can conclude that this type of system can be developed using agile methodologies.

### 2.2. Standards That Define the Stages and Processes of the Software Systems Life Cycle

**Figure 1:**
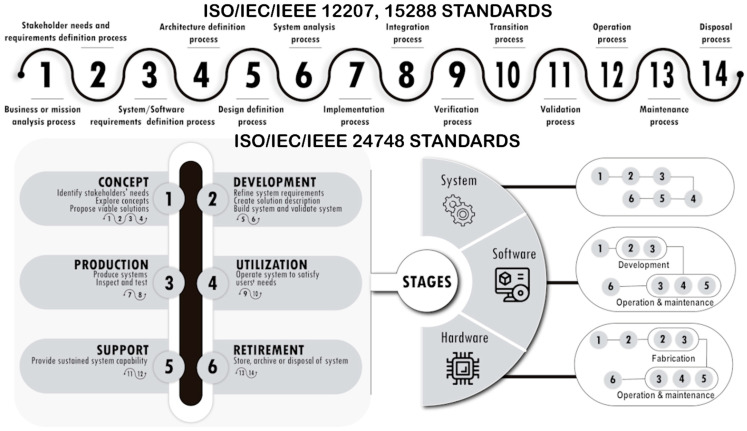
Summary of the stages and processes of the life cycle of software systems considered in the different reviewed ISO/IEC/IEEE standards [61,67,72,87,88].

ISO/IEC 12207:2017 [72] addresses software lifecycle processes, while ISO/IEC/IEEE 15288:2015 [67] addresses systems lifecycle processes. In turn, the ISO/IEC/IEEE 15289:2019 standard [61] proposes a common life cycle for systems and software engineering, based on the life cycle processes specified in the two standards. Similarly, the purpose of ISO/IEC/IEEE 24748-1:2018 [87] is to facilitate the joint use of the content of ISO/IEC/IEEE 15288 and ISO/IEC/IEEE 12207, providing unified and consolidated guidance on systems and software lifecycle management. On the other hand, ISO/IEC/IEEE 24748-3:2020 [88] provides guidance on the application of the software lifecycle process standard, i.e., ISO/IEC/IEEE 12207:2017. In addition, ISO/IEC/IEEE 24748-4:2016 [89] provides detailed requirements and guidance on the application of system lifecycle processes, i.e., ISO/IEC/IEEE 15288. Figure 1 has been prepared after reviewing the standards to show the life cycles and processes that these standards involve.

Methodologies are free to specify how to execute the stages and technical processes that the ISO/IEC/IEEE standards propose, their execution order, and even select which processes will be executed [67]. However, presumably, at least the following processes must be present in the lifecycle: (1) planning and requirements gathering, (2) solution analysis, (3) solution design, (4) solution coding and unit testing (construction), (5) integration and testing (implementation), and (6) operation and maintenance [64,90,91,92]. Additionally, all the work done in each of the activities carried out should be well documented. Therefore, the difference between the methodologies must be in the processes to be considered, in the way they are executed, and in their execution order [61,67,72,87,88,89,91].

### 2.3. IoTS Development Methodologies Based on the Agile Manifesto

These methodologies split the system to be developed into deliverable products, which in turn are organised into tasks that last from 2 to 4 weeks (called *sprint* in Scrum) [92,93]. This way of organising the work contrasts with other development methodologies, such as Waterfall, Spiral, RP, etc., that take the problem to be solved in its entirety.

#### 2.3.1. Guidelines for Project Risk Management

Agile methodologies treat risks indirectly, by establishing the characteristics of work teams. Thus, for example, Pico-Valencia et al. [94] present a methodology for small teams based on the principles defined in Scrum [95]. In this way, they try to make up for the lack of clear guidelines on the agile software development manifesto to specify stages or activities that deal with risks during the development of the systems. The authors consider that building small development teams and holding daily face-to-face meetings are two characteristics that can lessen the risks inherent when people are involved. The gradual delivery of deliverables can also reduce the risks inherent to the technology itself (its non-availability in the market, degree of dominance on the part of developers, etc.), and the budget (acquisition of components, staff training, and so on).

According to Abrahamsson et al. [5], the agile manifesto (and, consequently, Scrum) does not specify anything regarding software development processes. Consequently, Scrum could be considered a project management methodology rather than a software development methodology, since it does not specify the activities that must be carried out during the development life cycle, while it specifies how to manage the project lifecycle [44,70,71]. Muntés-Mulero et al. [96] incorporate risk analysis as an integral part of each sprint in the Scrum methodology.

In agile methodologies, which are the focus of this work, the processes that are specified will be carried out more than once, depending on the number of deliverables into which the system has been divided. The order in which they are executed may be different, depending on the design of the methodology. For example, tests in Scrum are performed at the end [92], while in XP they are written at the beginning of the development cycle, as stipulated by TDD (Test-Driven Development) [97].

#### 2.3.2. End-User Needs and Requirements Definition Process

One of the reasons behind the success of agile methodologies is the distinguishing feature of being able to make changes during the development of the system at any stage. Ideally, all requirements should be known and clear from the beginning of development, although this is not always the case. The elicitation of requirements is a problem known to developers [98] and causes developers to take the changes suggested by customers or end users throughout the whole development process.

For agile methodologies, ISO/IEC/IEEE 26515:2018 defines user stories, scenarios, and characters as tools for obtaining and analysing requirements, while use cases are proposed as a design technique [99]. For example, Scrum proposes to use user stories as a tool for acquiring requirements [7,8,92,100]. User stories are simple narratives that illustrate a user requirement from the perspective of a person or actor [98]. User stories must be thoroughly understood by developers to express system and software requirements. User stories are written in natural language. Therefore, they are unstructured tools and could be misinterpreted by developers [101].

It is imperative that there is a consensus among developers on how to properly obtain system requirements, as they are necessary to define the initial system architecture. Obtaining the requirements is complicated, and it is unlikely to obtain all of them at the beginning of the development of the system [102]. Therefore, in this scenario, it is foreseeable that there will be changes throughout the development of the system. Undoubtedly, the possibility of adapting to such changes, like agile methodologies can do, would be an important point to include in the guidelines of a methodology specifically designed for the development of IoTSs.

#### 2.3.3. Non-Functional Requirements

It is very important to reflect on non-functional requirements (NFRs) in the development of an IoTS. The security and privacy of data captured by sensors [103,104], the durability of power supplies (batteries) [91,105], the resilience of communications [105,106], and the intrusiveness of sensors [103,107], among other NFRs, must be considered at each stage of an IoTS development. Therefore, for example, there are research lines on different methods, protocols, and guidelines to guarantee data security and privacy [30,108,109,110], low energy consumption [110,111,112,113], or to integrate sensors with different levels of intrusiveness [114,115], among others.

Consequently, the importance given to NFRs in IoTSs could make agile methodologies inappropriate for their development, since requirements are elicited through user stories, due to the problems mentioned in Section 2.3.2. User stories could be used to identify NFRs when they are user needs [98], but according to Sachdeva and Chung [116], Scrum has no clear way to check whether NFRs are met or not. This risk can be reduced or even avoided if acceptance criteria are defined in detail, and NFRs are clearly described in user stories from the outset, relying on additional information and subsequent activities to this end, as advised by Pecchia et al. [98]. To ensure success in obtaining requirements, the writing of user stories (if such a tool is used to obtain requirements) should be carried out by both parties, i.e., developers and end users, who should also need to be involved in the development of the IoTS [96], to ensure that it will finally meet their needs and expectations.

#### 2.3.4. Number of Development Team Members

PMBoK [70,71] provides guidelines for managing work teams from small to large. However, popular agile methodologies have been thought to work with small teams of a maximum of 15 people, including the product owner [117]. For example, Kanban indicates a maximum of 14 developers [118], XP and Scrum, and a maximum of 11 members, with the scrum master and the owner of the product [117,118]. Regarding the roles of the team in Scrum, Kettunen, and Laanti [119] raise the need to create other roles additional to those defined in Scrum for larger software companies. Along the same lines, Morais dos Santos et al. [120] also raise the need to adapt Scrum for large software projects. In fact, they adopt the Scrum of Scrums (SoS) technique and add two new roles, called General Product Owner (GPO) and General Scrum Master (GSM). These new roles will serve to coordinate and assist other roles.

### 2.4. Modeling as a Key in IoTS Development Methodologies

From the perspective of software developers, IoTSs are mainly characterized by the heterogeneity of their components and the technologies they involve, in addition to the scarce processing capacity of each component [121]. For example, due to the lack of well-established standards at the hardware level, manufacturers often provide different implementations and/or operational features, which often leads to a heterogeneous software and communications platform (i.e., with different communication technologies and protocols) [34,122]. In addition, developers typically must deploy a shared set of functionalities across multiple and different devices [123,124,125]. Consequently, in that scenario, hardware heterogeneity leads to different source codes of the same software design, just to be able to support the different features of the underlying hardware.

Model-driven development methodologies can help address hardware heterogeneity. In fact, these methodologies were introduced with the aim of focusing on the design of a system’s functionalities, mainly at a platform-independent level of abstraction. Its main objective is to be able to obtain final implementations of the system with as little platform-dependent code as possible written by the developers.

The most important model-driven development methodologies known so far are Model-Based Engineering (MBE) [126,127], Model-Driven Engineering (MDE) [127], Model-Driven Development (MDD) [127], and Model-Driven Architecture (MDA) [127,128]. Although the similarity between them is easy to recognise, their difference lies in the importance they place on the models themselves, how they conceptually define “what is” a model, and how they are used to ultimately obtain an implementation of the system. In addition, they differ in the stages of development involved and the tools they propose to enable modelling [129].

Both MDD and MDA are guidelines to follow during the software development stages. The difference between them is that MDD can be written in any modelling language, while MDA is a standard specification that clarifies that UML (Unified Modelling Language) should be used as the main modelling language, while any transformation should be specified using the Query/View/Transform (QVT) language. MDA also specifies that models should be transformed following a descending abstraction level order, that is, from computationally independent models (CIMs) to platform-independent models (PIMs), and these two platform-specific models (PSMs) [127,128]. As expressed in the standard specification, MDA is an approach to software design, development, and implementation spearheaded by the Object Management Group (OMG) [130]. MDA provides guidelines for structuring software specifications that are expressed as models [131]. Thus, MDA aims to set aside the technical particularities of implementations, and instead focuses on “modelling” software solutions. On the other hand, it separates itself from requirement elicitation, assuming it as a preliminary work already completed at an earlier stage.

In MBE, neither model definition nor automatic code generation constitute key aspects of the development process. In addition, it considers models as plans that must be understood by programmers to write program code in a target programming language. Instead, MDE is governed by models, as models are expected to be defined to generate (semi-)automatically at least one partial codebase, or even other models from them [132]. This process is commonly referred to as model-to-text (M2T) or model-to-model (M2M) transformation. In addition, in MDE, models and transformations can be defined in any modelling language and can refer to different levels of abstraction.

## 3. Methodologies for Traditional IS Development Applied to IoTS Development

As mentioned above, methodologies devised for the development of conventional I’S have been used, and even some of them have been adapted for the development of IoTSs. To review the literature on this subject and analyse the existing proposals, the related articles have been retrieved and reviewed, consulting several scientific databases (ScDBs), namely, Web of Science (WoS) (https://www.webofscience.com/wos/alldb/advanced-search, accessed on 21 November 2022) and Scopus (https://www.scopus.com/search/form.uri?display=advanced, accessed on 22 November 2022), IEEE (https://ieeexplore.ieee.org/search/advanced, accessed on 23 November 2022), and ACM (https://dl.acm.org/search/advanced, accessed on 24 November 2022), to ensure that we consider the largest number of articles published on this research topic.

**Figure 2:**
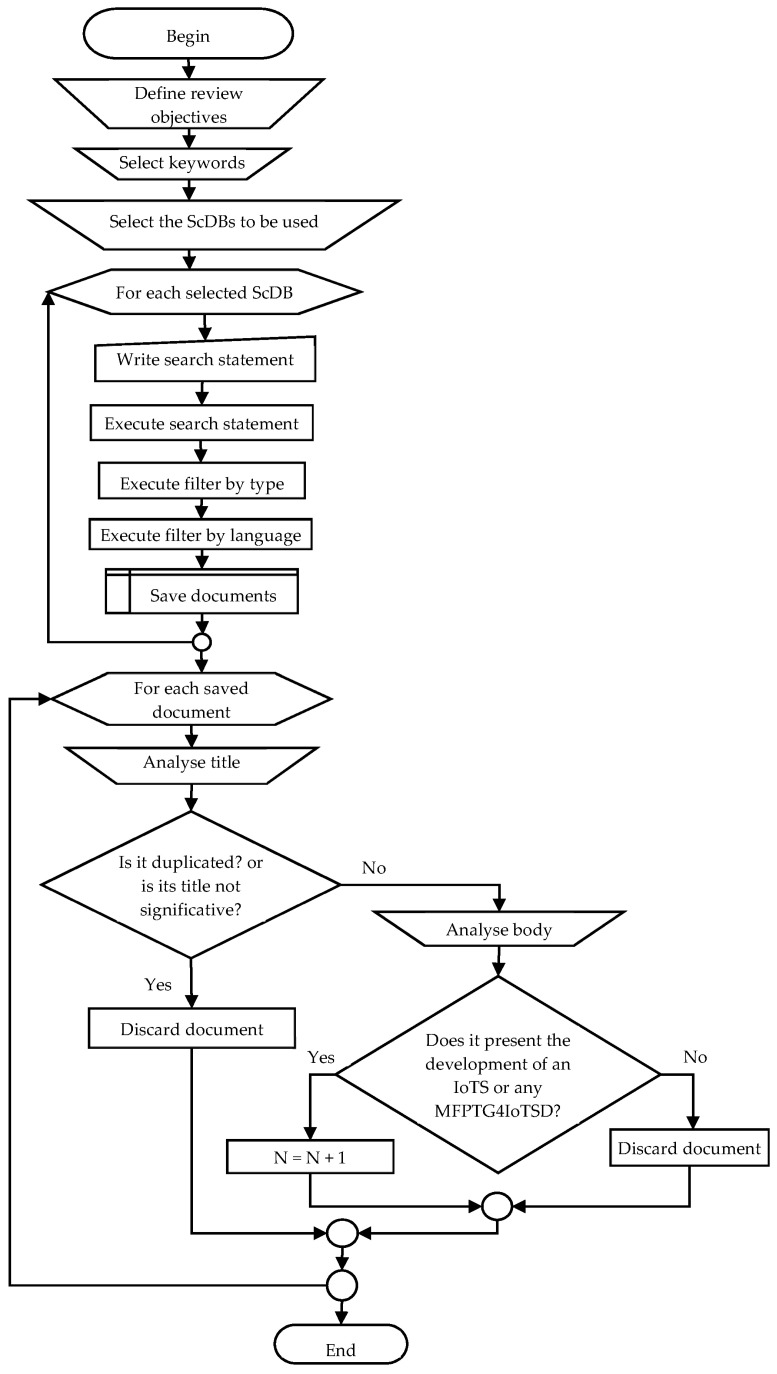
Flowchart of the state-of-the-art review process.

Figure 2 shows the flowchart of the procedure carried out for the retrieval of documents for the review of the state-of-the-art that is being presented in this article. It shows the processes that the authors had to perform manually and the processes that were executed automatically with the help of the tools provided in the ScDBs consulted. The objective of our search was to retrieve the documents in which some Methodology, Framework, Platform, Tool, or Guidelines for IoTS Development (MFPTG4IoTSD) is proposed, in addition to those in which the development of an IoTS is described, provided that the methodology applied to develop it is specified.

The search carried out was very extensive, as can be seen by consulting Table 1, which shows the search statements entered in each of the ScDBs consulted, as well as the number of documents initially retrieved and the number of documents resulting after applying each of the filters indicated in the procedure described in Figure 2. The execution of the queries was carried out on 14 October 2022, and alerts were registered in the databases so that the data of the new documents that meet the search criteria established for this work are sent to the email of one of the researchers (last revision on 22 December 2022). At the time of submitting this article, no new matches have been received.

**Figure 3:**
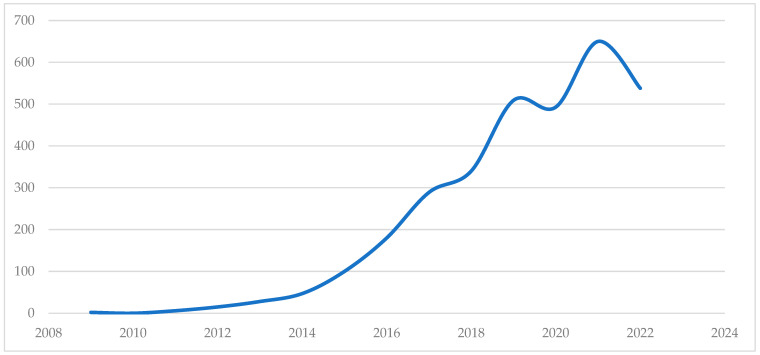
Year of publication of the documents selected for analysis.

Making use of the analysis tools of the WoS platform, Figure 3 shows the distribution in time of the jobs retrieved with our search sentence (3201 records) since the beginning of the popularisation of the term IoT in 2009 [133], having obtained only 2 works published in that year. The appearance of articles related to IoT is constantly growing, although in 2020 there was a decrease in the number of publications of research papers, probably due to the COVID-19 pandemic, which led to health problems and restrictions on the mobility of the population [134,135]. However, this growth resumed in 2021. It should be noted that from 2022 onwards, papers published up to the date of submission for publication of this article have been considered.

Of the filters applied (see Table 1 and Figure 2), the first two, that is, by title and language, were automatic filters. The *Type* column in Table 1 indicates the number of papers that meet to be peer-reviewed articles and published in standard publication sources, such as journals, conferences, book chapters, and books. The *Language* column shows the number of articles that have been written in English or Spanish (only one of the finally selected is written in Spanish). However, the filter by title was carried out manually. The *Title* column shows the number of articles whose title was considered significant for the present investigation.

Another of the manual filters, and the most important, was the filter for the information contained in the body of the document. Thus, to be considered a document, it must present: (1) The development of any IoTS, application or device, provided that its authors present evidence of having used any MFPTG4IoTSD; or (2) A development methodology, so that (2.1) the main objective of the authors of that MFPTD4IoTSD has been the design and construction stages of the corresponding system, or (2.2) the work presents some broader MFPTD4IoTSD, that is, it does not only specify the design and construction phases of the system.

**Figure 4:**
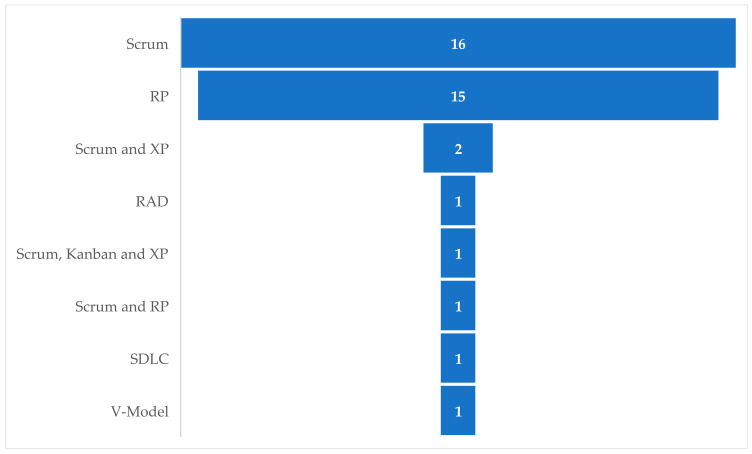
Number of IoTSs developed with methodologies designed for the development of ISs.

After the application of this last manual filter, 60 documents of interest for the present research were found, of which 38 documents present the development of some IoTS following a development methodology for traditional IS (see Figure 4), 8 papers present MFPTG4IoTSDs that address the design and construction phases of the software for IoTSs, i.e., they differ greatly from the life cycle presented in the ISO/IEC/IEEE 15289:2019 standards, and 14 documents present MFPTG4IoTSDs that can be considered to be within the ISO/IEC/IEEE standards mentioned. Documents presenting the development of any IoTS where the development methodology used is not clearly specified were not considered.

Figure 4 shows the frequency or number of times that methodologies devised for the development of conventional ISs have been applied to the development of IoTSs in the set of documents analysed in the study we have carried out. 

As can be seen in Figure 4, most of these IoTSs have been developed using the Scrum methodology [60,116,120,136,137,138,139,140,141,142,143,144,145,146,147,148], specifically in 42.11% of the IoTSs considered, with RP [149,150,151,152,153,154,155,156,157,158,159,160,161,162,163] being the other most used methodology, with 39.47%. In addition, Scrum has been used in combination with other methodologies in 10.53% of cases. So, for example, there are developments by combining Scrum with XP [57,58], or using a combination of Scrum, XP, and Kanban [164], or combining Scrum with RP [59]. The least used methodologies have been Rapid Application Development (RAD), V-Model [165], and SDLC (System Development Lifecycle) [166], representing 2.63% each, while Kanban has only been used for the development of IoTSs in combination with Scrum and XP.

**Figure 5:**
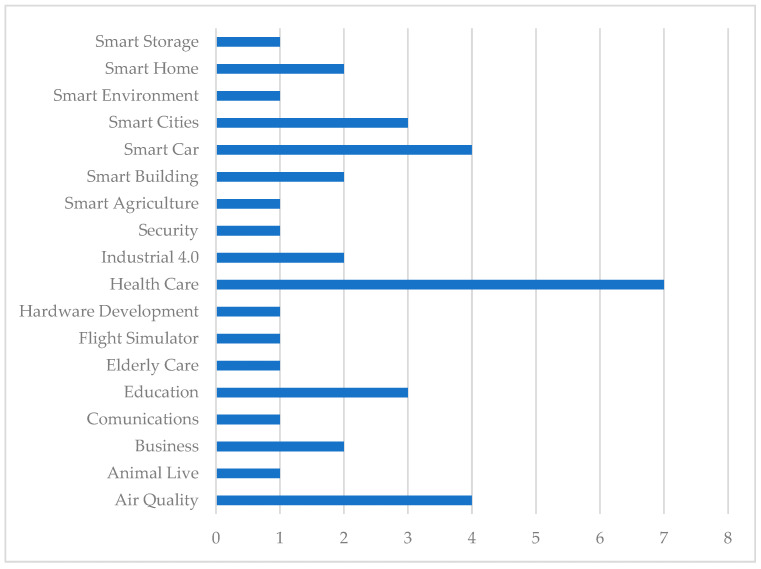
IoTSs application areas are developed with methodologies designed for ISs.

In the works represented in Figure 4, the areas or fields of application in which the IoTSs proposed them have been developed have also been identified. The results of this analysis can be seen in Figure 5, where it can be seen that the domain in which the development of IoTSs has been most formalised is Health Care, with 18.42%, followed by those of Smart Car and Air Quality, with 10.53% in each of them with respect to the total of the works considered for this analysis.

Although the growth of IoT and IoTSs has been dizzying in recent years, not everyone has access to the Internet yet. According to the World Bank [167], only 49. 72% of the population has access to the Internet. In fact, there are countries whose population with Internet access is below 25%. An even smaller percentage of people probably know what IoT is and how IoT can benefit them. One of the methodologies that has been successful when the client is not clear about their requirements, either because they do not know the use they can give to the existing technology, or because they are not very clear about the requirements of the system to be developed, is the prototyping methodology. Therefore, these data suggest that the use of prototyping for the development of this type of system will be a success and will be well-valued by customers. Moreover, the possibility of not continuing with the development of the system, whether due to lack of budget, technological issues (such as unavailable technology), or of any other nature, is another characteristic that supports the success of agile development.

### 3.1. Methodologies, Tools, and Frameworks Focused on the Design and Construction of Software for IoTSs

For this classification of documents, those with an MFPTG4IoTSD that do not specify the stages of the system life cycle were considered. These methodologies focus on the design and construction stages of IoTSs. In these MFPTG4IoTSDs, the design is mostly based on models and metamodels. For software construction, technologies are presented that are capable of automatically generating code in various languages. Most of them focus on generating code in C, C++, or variants, as they are the most popular type of languages on computer boards (controllers) used in the development of IoTSs. Another programming language in which they generate code is Java, and only one of the references found mentions the generation of code for the Node.js environment. Table 2 shows the main characteristics of non-traditional methodologies that address the design and construction stages of IoTSs. In addition, it shows the tools they have used or proposed to carry out their objectives.

To start the software design and construction stages, you must first go through the stages of analysing the needs of the stakeholders and the elicitation of system requirements, and then move on to the stage of analysing both system and software requirements. These stages are considered as very important stages in some works [168,169] (those marked with 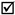 in the Requirements column of Table 2), being these stages, the providers of the information needed to continue with the design and construction. However, other works [170,171] (those marked with ~ in the Requirements column of Table 2) take them as resolved, putting a lower emphasis on them than in the previous works, and without specifying any analysis method or tools to be used. Moreover, the methodologies presented in other works [172,173] do not mention the requirements.

Table 2 shows other aspects of the methodologies and tools reviewed in this study. More specifically, it shows whether the requirements analysis is considered, or it is only mentioned, or it is not specified in the corresponding methodology, as well as their respective modelling languages, and the artifacts used to obtain and analyse the requirements, model and/or generate the code.

The contribution of Lekidis et al. [168] consists of an IoTS design flow based on MDE and SOA. These authors focus on models for IoT Wireless Personal Area Network (WPAN) systems. This proposal also supports the modelling and implementation of the application functions to their deployment in the IoT system. The steps specified by the flow are (1) translation for the construction of the application model, (2) translation for the synthesis of the OS/kernel model, (3) transformation for the construction of the system model, (4) code generation, (5) space state exploration, (6) calibration, (7) verification of statistical models, and (8) injection of failures. Design activities are supported by requirements verification and validation processes, facilitating system model refinement. This ensures compliance with NFRs related to application performance and efficiency, as well as functional requirements (FRs). Their work focuses on the Contiki platform, which uses a proprietary DSL (Domain Specific Language) that serves to write the REST services that run on that platform.

MDE4IoT [172] is a methodology based on MDE that is focused on modelling and generating the final product. This methodology does not mention the stages of planning, obtaining, and analysis of requirements, operation, maintenance, or deployment. The elicitation of system requirements is also not addressed by its authors. To achieve the transformation of models to executable artifacts, MDE4IoT leverages a combination of Domain-Specific Modelling Languages (DSMLs). Modelling is done from three points of view: (1) specific software application domain, (2) physical devices, and (3) both software and hardware of a specific application domain.

IOPT-Tools [174] allows control modelling for embedded systems through a class of Petri nets called “Input-Output Place-Transition nets”. IOPT works through a Web interface and allows you to obtain the control code from a single graphical model. However, if you want to communicate different controllers with each other, then you must manually write and adapt the generated code. Therefore, although it is a tool for IoT devices in general, it does not consider how to implement the interaction with other components of an IoTS or with the users themselves.

Brambilla et al. [170] present an approach for building mobile applications for IoTSs. This approach is based on an extension of UML, known as Interaction Flow Modelling Language (IFML), designed to express the content, user interaction, and control behaviour of the front-end of mobile applications. To model both the events and actions associated with IoT devices, new elements have been added to this set of graphical notations (IFML) to create models that visually represent the behaviour of systems in the face of user interactions. To define patterns that encompass the most common IoT use cases, the authors define content class models and interaction class models. The patterns addressed are specific to IoT, user interaction, and data synchronization. It is not specified in which programming languages the software code is generated. In addition, this proposal covers some limited areas of application, and even in those areas it is limited to only certain types of systems.

Harbouche et al. [169] propose an MDE approach that allows developers to derive a system design from the overall specification of their requirements. Their design methodology follows the top-down paradigm, and they bet on automatic processes for the derivation of the behaviours of the global requirements of the system towards a set of collaborative components to eliminate possible errors. Each level of abstraction is described using a specific metamodel. Therefore, the application of an MDE approach requires the definition of the appropriate metamodels and the corresponding model transformations.

Chauhan et al. [175] present a development framework that encompasses the following: the domain specification, application architecture design specification, architecture framework generation, domain framework generation, definition of a set of abstract user interactions, generation of user interfaces, and description of the implementation specification. Another of its considerations is the generation of the code of the programs that can be deployed in the devices themselves. For each of these aspects of the methodology, a language is defined that will be used by the different members of the development team.

ROOD (Resource-Oriented and Ontology-Driven Development) [176] is a methodology based on an MDA approach, although it is supported by MDE-based tools. ROOD is oriented to the development of intelligent spaces from two points of view: (1) of the contextual activities or behaviour of the resources, being these the sensors and actuators, and (2) of the intelligent object. It includes Environmental Context Models (ECMs) and Smart Object Models (SOMs). Among MDE-based tools, ROOD incorporates a UML profile known as Smart Space Modelling Language (SsML). This methodology presents 3 main stages, namely, the first stage, ECM, which is related to the MDA CIM. The second stage, SOM is represented by the MDA PIM, and finally, the PSM. At each stage, Corredor et al. [176] address the verification of model consistency and semantic consistency from the point of view of ECM or SOM with the respective viewpoints, and according to domain concepts on the knowledge base. Although ROOD has been considered a methodology that is not in accordance with ISO standards [61], it clearly exposes the work that each professional must do along the development process, including analysis, modelling and implementation.

Finally, ELDAMeth [171] is a simulation-based methodology for Distributed Agent Systems (DASs), which allows rapid prototyping based on visual programming, validation, and automatic code generation for DASs based on the Java Agent Development (JADE) framework. ELDAMeth is an iterative development process for DASs that encompasses the stages of low-level design, simulation-based validation, and JADE-oriented implementation. However, it does not present information on how the authors approach the stages of planning, elicitation, and analysis of requirements, in addition to the integration, operation, and maintenance stages.

All the development methodologies presented in this section are focused on the design and construction (automatic code generation) stages, based on a model transformation approach to obtain the IoTS software code. However, some of them [168,169,172,174,175,176] are specifically based on the transformation of models, while others [170,175] are based on patterns, and another [171] is based on agents. 

### 3.2. Methodologies Designed for IoTS Development in Accordance with ISO/IEC/IEEE Standards

Fifteen documents were found presenting some development methodology for IoTSs, out of the several documents obtained from the ScDBs consulted. Table 3 shows the methodologies found. The analysis carried out on these documents was oriented to the formality of the methodologies, that is, they present the stages or processes of systems and software engineering, as well as the tools that they recommend using to obtain the deliverable product at each stage. In a quick review of the selected documents, it has been possible to determine that most of them comply below 50% of the stages that ISO/IEC/IEEE standards establish in the life cycle of systems/software [61,67,72,87,88,89]. For this reason, we have reviewed the literature to determine which stages should have an appropriate life cycle and if there is a consensus in the literature on this matter. Common stages in systems life cycles are (1) planning, (2) analysis (of requirements and software/system), (3) solution design, (4) solution coding and testing (construction), (5) integration and testing (implementation), and (6) operation and maintenance [64,90,178,179]. Therefore, we have considered that these stages are the ones that a methodology should specify.

The analysis of the documents presenting these methodologies was able to determine the degree of compliance (expressed with an adequate clarity in the corresponding paper) of these 6 stages, whose score was as follows:

- Proper compliance. The authors have addressed all stages of the software/system life cycle, recording evidence of this, such as explaining what it consists of, and the tools to be used, among other aspects (✓).
- Incomplete compliance. The authors, although not specifically naming some stages, name some activities, tools to be used, or other aspects of those missing stages. For example, they mention the use of use case diagrams, class diagrams, or software generation from models, among others (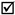).
- Legacy compliance. The authors do not explicitly name some stages because they are part of or already present in the approaches on which they are based. For example, in some cases, they mention that they are based on the fundamentals of SCRUM, which suggests that the planning stage is carried out (**±**).
- Inadequate compliance. Works in which the importance of some stage is mentioned, but without giving more detail on how to carry it out (**~**).
- Non-existent compliance. Works in which their authors do not mention activities of some stages or even do not mention certain stages (✗).

The *Other* column refers to other additional stages included in the analysed methodology, even as a consequence of having divided one or more of the 6 stages considered, as long as this is in accordance with the life cycle of the software/system presented in the ISO/IEC/IEEE standards [61,67,72,87,88,89] The results of this analysis are summarised in Table 2 and detailed below.

RASPSS [180] is a methodology for the design and implementation (construction) of intelligent health service processes in the context of industrial interconnection and iterative design of rehabilitation assistance devices. This methodology, in addition to being directed to a particular type of system, guides directly to the construction stage of this type of system. The architecture it presents clearly shows the importance of interaction with the user. It addresses in detail the creation of IoT devices, including providing them with the artificial intelligence techniques necessary to achieve their objectives.

The work of Schauer and Falas [181] focuses on the development of atomic services. Services are deployed in a service repository according to the types of computing resources that are available. That repository has been designed to provide two functionalities: (1) the list of services with the appropriate descriptions; and (2) pluggable Docker images ready to use in different architectures based on atomic services. Its architecture is based on a repository of Docker image forms and controlled by the service orchestration process. The atomic services are developed following a life cycle with the stages of design, development (a stage of this methodology), deployment, and execution. In addition, IoTSs are treated as complex systems and are carried out through service composition. A third issue addressed refers to the components related to computational resources (processing, storage, and so on), and communications (protocols, technologies, and so on). This methodology is focused on the stage of IoTSs construction and is closed to the technologies. These may be the reasons why the planning, integration, and maintenance stages are not mentioned. Other activities are assumed to take place in what they call the development stage.

TDDM4IoTS [37] is a methodology based on the 4 values and 12 principles of the Agile Manifesto. It considers 11 stages and specifies, in an acceptable way, the resources and tools to be used in each of the stages. It is the most attached to the software/systems life cycle raised in the ISO/IEC/IEEE standards [61,67,72,87,88,89]. TDDM4IoTS raises the stages in a distinctive way, involving those aspects of IoTSs that differentiate them from the traditional ISs, except that it does not address those activities that involve the provision of AI techniques to an IoTS, as well as the withdrawal of the IoTS once it cannot fulfil the functions for which it was designed, or its maintenance is unfeasible.

Another methodology based on the principles and values of agile methodologies is the one presented by Pico-Valencia et al. [94]. This methodology is focused on the construction of IoTSs. It presents the requirements elicitation stage by collecting the global requirements, being the work team who collects these requirements from users, clients, and other stakeholders. The design stage consists of, once these requirements have been defined, carrying out a set of tasks linked to very specific agents. More specifically, an exploration process of the IoT infrastructure is carried out, where the objects connected to the network are modelled as Linked Open Agents (LOAs). In addition, tasks related to the infrastructure itself are carried out (interception of messages, adding new messages, workflow execution, and agent discovery). The construction and integration stages are presented as a macroscopic level stage in which the microscopic level LOAs are integrated and coordinated within a single network. Moreover, its authors have designed this stage to meet the requirements of LOA interaction, collaboration, coordination, data processing, user interaction and intelligent behaviour. It should be noted that this methodology mentions the use of tools that the developer can use in the analysis and design stages. However, there is no evidence of the planning, implementation, operation, and maintenance stages.

Among the contributions of Gogineni et al. [86] is a methodology for the development of IoTSs that follows the V-model XT. In it, the verification and validation of the requirements, functionalities, and principles governing the system is the focus of its considerations. Other important stages are the requirements elicitation, design (in some respects), integration, and testing stages. However, they do not present evidence to address the operation and maintenance stage. 

INTER-METH [65] can be considered an iterative methodology that defines the six sequential stages of development: analysis, design, implementation, deployment, testing, and maintenance. Each iteration can be geared towards improving individual stages, a set of successive stages, or the entire process, thus improving adaptability to new requirements. INTER-METH is a methodology adapted from the traditional waterfall methodology, differing in that it divides the problem into subproblems to ensure successful development. INTER-METH, being based on the waterfall methodology, is supposed to meet each of the characteristics of its base methodology. However, its authors do not present tools, guidelines, activities, or tasks typical of IoTSs, such as hardware deployment.

Usländer and Batz present SERVUS [102] as an IoTS development methodology aimed at solving interoperability challenges by adopting a service-oriented architecture based on the Industrial Internet Reference Architecture (IIRA 4.0). SERVUS addresses requirements elicitation and analysis, as well as the analysis and design stages. To obtain the requirements and their analysis, they recommend user stories and use cases as the main artifacts for this stage. However, there is no supporting evidence for the software construction, deployment, or operation and maintenance stages.

Sosa-Reyna et al. present a methodology with two approaches, i.e., with the MDD [124,125] and MDE [123] approaches. This methodology establishes the following stages of development: (1) Analysis of business requirements, (2) definition of the business logic, (3) design of the integrated services solution, (4) generation of the technological solution, and (5) model transformation methods. They leverage the MDE guidelines and use languages such as Unified Modelling Language (UML) to specify business requirements in stage (1), and Business Process Model and Notation (BPMN) for the definition of the business logic in stage (2). The result of stages (1) and (2) is a PIM. Subsequently, a more refined model is obtained following an SOA approach in stage (3). To obtain a platform-specific code in stage (4), two steps are performed: First, a PSM is derived from the PMI, and second, the PSM is transformed into a code. This is one of the IoTS development methodologies that presents and defines all the basic stages for the development of this type of system. However, its authors do not consider how to develop user interfaces. Moreover, they present a set of tools for capturing and analysing FRs of IoTSs, but NFRs are not considered.

The SEM methodology [182] is based on metamodels. The modelling is done from two points of view: from the functions that the system must fulfil and from the data with which it works. It presents 3 stages: the Requirements Analysis stage, in which the provided metamodel is used to obtain the model in the Design stage, and finally, the System Implementation stage. SEM is another of the methodologies focused on the construction of IoTSs, so it focuses on the necessary aspects to obtain the final product with the requirements demanded by the users. However, it only mentions important stages, such as planning, requirements elicitation, implementation, and operation and maintenance of the IoTS. Moreover, it is a specific methodology for a specific domain.

Arrowhead [183] is presented as a framework. The design, development, and verification methodology for each service, system, and system of systems within the Arrowhead framework supports that these can be implemented, verified, deployed, and executed in an interoperable manner. Arrowhead helps in the construction of IoTSs from the perspective that the system can be built by integrating other systems. Although its authors mention the stages of the software/system life cycle a lot, they do not present evidence to guide developers to carry out their activities.

Costa et al. present IDeA [184] as a methodology for the development of IoTSs, based on model-based systems engineering (MBSE). The method provided is based on existing standards to which activities considered the most relevant for the design of IoT applications are added. IDeA provides high-level abstractions through metamodeling as a possible solution to the (hardware and software) heterogeneity problem. In addition, their IoT application design method is a multidisciplinary method, in which all the stakeholders involved in the process participate. They apply the ISO/IEC/IEEE 15288:2015 standard, from which they implement the systems’ life cycle processes, although they do not specify all their phases. Although IDeA refers to the ISO standard, it does not provide guidelines for its application. In addition, there is no evidence that deals with the planning stage and, being a methodology based on metamodels, forces developers to stick to them.

The methodology proposed by Cicirelli et al. [182] is based on both functional (functions and services) and data (data sources, attributes, and relationships) metamodels, and is focused on the design of IoTSs. Its authors assume the requirements elicitation as a preliminary stage and focus directly on the system modelling. By being based on metamodels, it raises stereotypes for: (1) the environment in which the system is going to work, (2) the functionalities that an intelligent environment can offer, which can be atomic or composite. In the metamodels, this methodology specifies the processes that will make the environment an intelligent environment. To use a metamodel, the problem must be aligned to the metamodel to be applied in the solution. The methodology is clearly oriented towards the design stage, but there is no evidence that this methodology addresses requirements elicitation and analysis. It also does not address the IoTS development planning or final stages, such as implementation, operation, and maintenance. In addition, the stage of construction or obtaining the final product is not clear.

Patel and Cassou [78] focused on the roles of team members to try to solve the problems of heterogeneity of technologies with which IoT applications can be implemented. Domain experts and software designers oversee the system analysis and design stage activities. Application programmers and device developers are engaged in the system construction and testing stage. Finally, network administrators install the application on the system in question, which fits with the implementation and deployment stages of other methodologies. This methodology does not address the planning, operation, and maintenance stages.

Fortino et al. [185] propose a metamodel-based engineering approach for the systematic development of smart objects (SO). The analysis stage deals with the modelling of relevant aspects of SOs using a metamodel. The design stage tries to model the functional components of the system, their relationships, and interactions using the smart object model, based on an ELDA framework [177] and an ACOSO platform [186]. To support the implementation stage, the metamodel of smart objects based on ACOSO has been specialised with respect to the JADE platform [187], resulting in the JACOSO metamodel [188]. This metamodel highlights the components of the JADE platform (people, their behaviour, and messages). Other elements of the metamodel represent the tasks to be respectively carried out by the person responsible for configuring the system, the communication manager, and the device manager, as well as the user-defined tasks that are included in the inference rules, which control the behaviour of smart objects. To use this metamodel, the developer must master the frameworks and platforms named above.

AMG [38] is an IoT application development methodology based on SOA consisting of three steps: (1) definition of abstractions, (2) modelling, and (3) code generation. AMG is based on a bottom-up approach since it starts from concrete models (concrete services) to obtain abstract services. The abstraction process starts from the descriptions of the services, in such a way that first the necessary graphic representations are obtained and then the source code is obtained.

Some authors, such as Gogineni et al. [86], Wang et al. [180], and Fortino et al. [185], consider requirements analysis as part of the system analysis stage, which constitutes the reason why they do not address the requirements elicitation. However, other authors, such as Guerrero-Ulloa et al. [37], Usländer and Batz [102], and Sosa-Reyna et al. [123,124,125], separate these stages very well and give them the importance they require, since the system requirements must be clear enough from the very beginning so that the development of the IoTS is not delayed. It could be concluded that the lack of consideration of the planning stage is widespread since only Guerrero-Ulloa et al. [37] expressly specify it, while Gogineni et al. [86] only give hints of its consideration, and Fortino et al. [65] only let us guess its consideration, since its proposal is based on waterfall methodology, which does contemplate this stage.

Another stage not mentioned by most researchers who have designed development methodologies is the joined operation and maintenance stage (i.e., a unique stage combining operation and maintenance activities). Varga et al. [183] and Guerrero-Ulloa et al. [37] present the maintenance stage as part of their respective methodologies. However, Wang et al. [180] and Pico-Valencia et al. [94] address this stage briefly. It can be assumed that Costa et al. [184] address this stage due to the approach its methodology (IDeA) relies on. Although Schauer and Falas’ [181] proposal is considered to address design, the work is focused on presenting an adaptive architecture for IoTSs.

Among the methodologies that have addressed the obtaining of software code is AMG [38], Schauer and Falas’ [181] one, and SEM [182], particularly in the modelling and/or obtaining of software for the IoT device, ignoring the applications that will serve for the interaction between the user and the IoTS [38,174]. Although Wang et al. [180], Pico-Valencia et al. [94], Schauer and Falas [181], and Fortino et al. [185] address and mention user-centred design, they do not present evidence of addressing the construction of end-user applications. The authors mentioned focus on obtaining the hardware component of the IoTS and the software for its configuration.

## 4. Other Proposals for the Development of IoTSs

There are many contributions oriented to facilitate the development of IoTSs. Some of them were already analysed in previous sections. Table 4 presents the main features of additional platforms, frameworks, and tools for the development of IoTSs.

Pawar et al. [154] propose PrIoT, a development framework that proposes to group third-party solutions to meet certain goals. The framework is composed of three modules to try to integrate the different components that make up an IoTS. PrIoT-core makes the work of the developers independent of the devices and the details of the communication protocols. PrIoT-Lang deploys a device-independent programming interface. PrIoT-API captures the most practical IoT scenarios in a limited way. PrIoT-Test allows to specify metrics to test the validity of the results obtained from the prototype. PrIoT-DB allows us to select the necessary hardware either from a specific or generic supplier and include libraries in the project, for example, to enable information exchange with other devices. The components are configured through a file. PrIoT-UI introduces a high-level graphical interface to support developers.

Cai et al. [191] propose a mobile application deployment framework based on MDA. Development patterns based on semantic reasoning are provided to target the development of Cloud of Things applications (CoT) [194] in a configurable and adaptable way. They also provide a metamodel with multi-view business components and service components for exchanging models. They follow the MVC (Model-View-Controller) pattern to transform business models into a service component to configure cloud services.

COMFIT [192] is an Integrated Development Environment (IDE) based on MDD and Cloud Computing. COMFIT consists of two modules: (1) a development module for designing IoTSs using adaptive models of high abstraction; and (2) a set of web-based management and execution applications, capable of generating code aimed at the Contiki and TinyOS platforms from the models obtained in the design stage. It is worth mentioning that COMFIT does not address how to develop applications for the end user.

EDG [193] is a methodology for the generation of integrated designs. To do this, the user is required to specify with simple requirements their embedded software. From that specification (hardware-independent code), a synthesised final circuit diagram is produced, a list of required materials and the firmware necessary for the device to meet the requirements. Finally, the hardware configuration diagram allows us to develop the physical device and write the hardware-dependent code.

TinyLink [190] is a tool that follows the EDG methodology [193]. TinyLink is aimed at developers with no experience in embedded systems, which may have both hardware and user constraints. TinyLink seeks to optimise hardware use through a set of APIs to allow developers to carry out a bottom-up development process, in contrast with traditional tools, which usually follow a top-down approach. In addition, TinyLink can generate a hardware-dependent code. Similarly, Autolink [189] is a tool based on TinyLink [190]. In addition to generating TinyLink’s functional solutions, Autolink addresses NFRs such as estimating the useful lifetime of the device’s hardware components, execution time, and optimising battery use.

All these works support developers when it comes to having the software code for the configuration of the IoT hardware. However, there is no evidence that it has been addressed how to obtain the software code for applications that will serve as a means of interacting with the end user. In addition, the presented tools need previously elicited requirements as inputs for the design stage and subsequent code generation.

## 5. Architectures for IoTSs

The architecture of any system serves as a guide in its development process. Thus, it is important to know the architectures that the researchers have proposed to develop IoTSs, as well as if the methodology they present for the development of IoTSs is based on some architecture, and which is the most frequent. Among the most frequent architectures are layered and service-oriented architectures. In addition, some authors [123,124,125,170] present a combination of both types, while other authors [78,94,139,169,195,196] present their own architectures to give solutions to different types of IoTSs.

### 5.1. Layered Architecture

**Figure 6:**
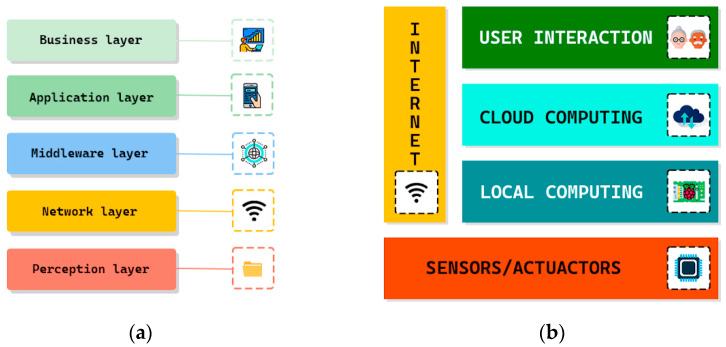
Support architectures of MFPTG4IoTDs for IoTS development. (**a**) Vashi et al.’s architecture [197]. (**b**) Guerrero-Ulloa et al.’s architecture, adapted with permission from Ref. [22]. 2022, Gleiston Guerrero-Ulloa, Carlos Rodríguez-Domínguez, Miguel J. Hornos.

Some existing IoTSs have been developed following a layered architecture, such as the one presented by Vashi et al. [197], which consists of five layers (see Figure 6a). Guerrero-Ulloa et al. [22] present a very similar architecture that also consists of five layers, as shown in Figure 6b. As can be observed, there are similarities between both architectures. Other similar architectures only have four layers [65,151,198]. The difference between these last architectures is the order of their layers. In some of them [65,151], their layers are (1) device, (2) network, (3) middleware, and (4) application, while in the other [198], its layers are: (1) perception, (2) middleware, (3) services, and (4) applications. Consequently, the lower layers (1) and the upper layers (4) have the same objectives, while the second and third layers are exchanged regarding the ones of the other two architectures.

Unlike the architectures of the works, the RapIoT toolkit [162,163] and the Mobile Health Platform [160] are based on a three-layer architecture, where two of the layers fulfill similar functions (device management and applications) in both architectures, only being different in the third layer (communications). In the former [162,163], this layer fulfils the functions of a cloud service, which allows data storage and integration with third-party services, while in the latter [160], it consists of the middleware (application server) and the web application that interconnects the various objects of the physical layer with other actors (health professionals, hospitals, and other systems) [162,163]. RapIoT layers [162,163] are named as an *embedded layer*, *gateway layer,* and *server layer*, and in the Mobile Health Platform [160] as a physical-objects layer, network layer, and health portal. The third layer performs very similar functions to the middleware layer of Fortino et al. [65] and Sharma et al.’s [151] architectures, the services layer of Qiang et al.’s [198] architecture, and the cloud processing layer of Guerrero-Ulloa et al.’s [22] architecture. 

Nugra et al. [57] present an IoTS to manage urban traffic, implemented following a three-layer client/server architecture. The *client* layer consists of applications for end users, whose input data are basically provided by Pentaho BI. The *business* layer is made up of APIs that provide weather data, in addition to the *Pentaho BI server layer* and the web application. They use two database managers: MySQL, which stores data captured by sensors and weather forecasts, and PostgreSQL, which is where data is copied, from time to time, to form OLAP cubes to allow data analysis.

ELDAMeth [171] uses ELDASim, which is an ELDA simulation environment, which is based on a four-layer architecture: (1) A configuration layer allows setting up its components through the MAS (Multi-Agent System) simulation module; (2) an agent layer provides adaptations of ELDA agents on agent servers; (3) the platform (i.e., the agent servers, the network that interconnects them, the signalling messages between agent servers and various types of systems, and the coordination infrastructures to fully support the distinctive multi-coordination feature of the ELDA model), which defines a distributed infrastructure consisting of a network of agent servers supporting ELDA agents; and (4) an engine that provides the key mechanisms for the simulation of discrete events of general purpose systems.

The methodology presented by Usländer and Batz [102] is based on IIRA v1.9 [199]. It defines a three-level scheme: (1) The edge level collects data from edge nodes, using the proximity network. The architectural features of this level, including the breadth of distribution, location, the scope of governance, and nature of the proximity network, vary depending on the specific use cases. (2) The platform level receives, processes, and forwards control commands from the enterprise level (explained below) to the edge level. It consolidates processes and analyses edge-level and non-edge data flows, as well as provides device and asset management capabilities. It also offers non-domain-specific services, such as querying and analysing data. (3) The enterprise tier implements domain-specific applications, decision support systems, and provides interfaces to end users, including operation specialists. This level receives data streams from the edge and platform levels, and issues control commands directed at both [200].

In the work by Lekidis et al. [168], the system design is specified in a domain-specific language (DSL), which they use to maintain the match between the automatically generated BIP (Behaviour, Interaction, Priority) model and the application code. BIP is a language with formally defined semantics for constructing executable models of mixed software/hardware systems (SW/HW). The BIP model is based on the standards of the WPAN architecture. This mixed architecture consists of four layers of abstraction, where the lower layer (1) is defined by the abstraction of the hardware architecture, the upper layer (4) is the abstraction of the software application, the third layer is defined by the operating system, and the second layer is defined by the network stack and device drivers.

### 5.2. Service-Oriented Architectures

SOA is used to build software systems from composite, heterogeneous, and autonomous software units, called services. In addition, service composition is a common approach to the development of complex software systems. Software systems and applications in turn are also becoming services. We call this service-based systems or applications [38].

In some of the works considered in this review, Sosa-Reyna et al. [123,124,125] propose an architecture based on SOA, consisting of four layers: (1) object layer (hardware objects available on the network), (2) network layer (wired, wireless or mobile connection infrastructure), (3) service (creation and management of required services), and (4) application layer (responsible for delivering applications to IoTS users). This service-oriented architecture supports development methodologies with two different approaches: MDD [124,125] and MDE [123].

Another work with this type of mixed architecture is defined by Brambilla et al. [170], with basically 3 layers: the client or front-end layer, the communications layer or API Gateway, and the server or back-end layer. This last layer is in turn defined by microservices, which provide information for user management, group concepts related to both the organisational structure of the actors and the definition of things and allow clients to access both data values and graphic resources.

The method for the development of IoTSs proposed by Sulistyo [38] is SOA-based. In the first instance, this author considers the existence of concrete services to abstract the abstract services to model the system in question. After obtaining the model, it generates the code for the new service-based application. This type of architecture (SOA) significantly reduces the complexity in the design of a heterogeneous system, such as an IoTS [168]. Another SOA-based work is the Arrowhead framework [183], where operations on different resources can be grouped into different services. In Arrowhead, a resource could be a temperature sensor or the energy consumption reading of an energy meter.

### 5.3. Other Types of Architectures

MDE4IoT [172] is based on the MARTE architectural model [201], which includes 3 packages: (1) MarteFoundations, which defines all the basic concepts required for the analysis and model-based design of real-time and embedded systems (RT/ESs); (2) MarteDesignModel, which covers from requirements capture to requirements specification, design and implementation (V-cycle development process [202]); and (3) MarteAnalysisModel, which defines specific model abstractions and relevant annotations to be used by external tools. Therefore, package (1) defines general concepts for quantitative analysis techniques, which are extensible to support new RT/ES UML model analysis techniques, while packages (2) and (3), respectively, focus on programmability and performance analysis.

The ROOD architecture [176] is based on MDA. It includes four levels of abstraction, ordered from the highest to the lowest level of abstraction as follows: (M3) Meta-Metamodel layer, which serves to establish the basis for different metamodels; (M2) Metamodel layer, where DSLs are specified to define models at the M1 level; (M1) Model layer, where system models are defined; and (M0) Instance layer, which contains instances of data for a given platform.

The COMFIT architecture [192] includes two modules: (1) Application Development Module (ADM), and (2) Application Management and Execution Manager (AMEM). ADM includes PIM and PSM models, as well as M2M transformations and code generation templates to use M2T transformations. On the other hand, AMEM includes: (1) the Interface Manager component, which is directly connected to ADM and is responsible for providing the functionality of uploading the generated code to a server hosted on the Cloud; and (2) the Execution Manager, which is connected to both the Testbed Manager and the Compile Manager and deploys the services that are released through the Interface Manager.

The framework proposed by Cai et al. [191] is based on software architecture for mobile service development, consisting of three modules: (1) an information module for device encapsulation, which supports multiple business modelling views; (2) an ejection environment, used to configure the application environment and the execution rules of a business application; and (3) a resource repository for information configuration, which is designed to connect the information modeller and the execution environment.

In the SDG-Pro framework architecture [203], components are classified according to their Internet connectivity: Edge or Cloud components. Edge components are sensors, actuators, and communication devices. Communication devices are connected to Cloud components through software-defined gateways that are part of Cloud components. In addition, as part of the Cloud components, we find the Information Technology components for data storage, processing, and intelligent analysis.

Another component-based architecture is the one presented by Schauer and Falas [181]. The first component is the IoT system builder. The second component is that of computational resources or a server, which is the one that interacts with feeding or receiving data from the other components. The server executes business logic when requested by Docker Engine clients and has the monitoring tools to interact with the supervisor component. The third component consists of the Docker Engine, which contains the technology for message exchange (RabbitMQ server). The fourth component is the IoT systems supervisor, which monitors resources and detects system failures. This last component interacts with the first one to update the monitored system according to what is measured/detected by the sensors.

The SEM methodology [182] is supported by an architecture based on models focused on software construction. Its authors present a functional metamodel and a data metamodel. Another methodology that is supported by this type of architecture is IdeA [184], where the IoT application engineer breaks down the system into functional components that interact with each other to meet system requirements. IDeA models expose devices as services using port notation. These services will be used by application engineers to create information views [184]. In this same line, we can mention Fortino et al.’s proposal [185], which is based on metamodels for smart objects as a very high-level metamodel that specifically exposes static and dynamic characteristics of smart objects. An ELDA-based metamodel specialises in the high-level smart object metamodel, providing the functional components of the system, their relationships, and interactions. And the ACOSO-based metamodel is a middleware specifically designed for the complete management of cooperating and agent-oriented smart objects.

Chauhan et al. [175] propose a publish-subscribe architecture in which sensors act as publishers. Computer services are subscribers that make actuators execute the corresponding actions (changes in the environment, notifications to end users, and so on) whenever new data is received.

The architecture presented by Harbouche et al. [169] is a wireless body sensor network architecture based on a mobile data collector. The architecture presented by Alvear-Puertas et al. [204] is similar. In fact, both are oriented to explain the operation and deployment of the system. They also provide feedback to the users about the outcomes of the requirements analysis stage. On the other hand, Wang et al. [180] present an architecture that can guide the development of an IoTS and understand how it works. In some works [37,86,174], the architecture of the IoTSs presented by their authors has not been addressed, only focusing on explaining its operation.

## 6. Conclusions

We have presented a comprehensive study on the methodologies as well as frameworks, tools, and architectures that could be applied to develop IoTSs. The main conclusions of the analysis carried out following the review of the existing proposals so far are set out below.

This article has discussed important aspects and steps that existing methodologies address or that a methodology for IoTS development should address, and these outline guidelines for implementation. An important aspect that differentiates IoTSs from other ISs is that they are made up of objects (*things*) that interact autonomously with each other, considering people as other objects or *things* of the system. To do this, IoTSs need to rely on sensor/actuator networks and efficient wireless communications [34,35]. Consequently, for the development of an IoTS, it is important to analyse existing and available technology, and even develop the necessary devices, if possible, to help meet not only FRs but also NFRs [32,35].

Model-based approaches, i.e., MDE, MDD, and MDA, are among the most widely used methodologies for IoTS development. However, very few of these methodologies deal with the elicitation and analysis of requirements, and except for one of them, no other deals with the maintenance phase, and none the withdrawal phase. Other aspects that most of the methodologies reviewed do not address or do not mention are those related to NFRs. The success of applying these approaches may be because they help to solve the technology heterogeneity problem involved in IoTS development.

We consider that the software development process should cover each of the stages/phases of the system life cycle, from the elicitation and analysis of requirements to its dismantling. However, with this type of system, as in others in some cases, the client is not clear about the requirements at the beginning, so it is considered that developers must present early prototypes. For this, model-based approaches must be present for the generation of software quickly, and therefore also RP. In addition, RP and agile methodologies are the most widely used in IoTS development. Therefore, SE researchers must design a methodology for the development of IoTSs that collects the best practices of model-based approaches (i.e., MDD, MDE, and MDA), PR, and the 4 values and the 12 principles of the agile manifesto on which agile methodologies (such as Scrum and XP, among others) are based.

None of the IoTS development methodologies proposed in the literature and pre-sented in this document have been used by developers or researchers other than their own authors. Most documents that present the development of some IoTSs apply Scrum as the only methodology, and some of them combine it with others, such as XP, RP, and Kanban.

The development frameworks used for IoTS development are mostly concerned with modelling and code generation for IoT devices, not addressing the generation of applications that offer an interface with the end user. We think that these development frameworks should also provide support for the development of such applications. An important advance in this area would be to create a tool that facilitates the work of IoTS developers in such a way that it becomes universally used by all of them. To achieve that goal, said tool should consider the functionalities of some of the tools presented in this review and add others that none of them currently have.

The most common architectural style in all the works that have been reviewed is the layer-based one. Usually, data processing occurs from the lower layer, known as the physical, perception, or sensors/actuators (among other names) layer, to the highest one, known as an application, user interaction, user, or cloud processing (among other names) layer, and the interconnection must be present between all its layers. The architecture serves as a guide to support the work of developers to fulfil the FRs and NFRs of the system, and therefore to obtain quality software.

## Figures and Tables

**Table 1:** Search statements for each ScDB were consulted and a number of results were consulted after executing them and after applying the corresponding filters. As usual, the wildcard character “*” is used to indicate that it could be substituted for any string (0 or more characters) at that place in the query.

ScDB	Search Sentence	Results	Filters		
			Type	Language	Title
ACM	Keyword:((IoT OR “Internet of Things”) AND (“develop* method*” OR “design* method*” OR “construct* method*” OR “implement* method” OR “develop* framework” OR “design* framework” OR “construct* framework” OR “implement* framework” OR “develop* tool*” OR “design* tool*” OR “construct* tool*” OR “implement* tool*” OR “develop* guidelines” OR “design* guidelines” OR “construct* guidelines” OR “implement* guidelines” OR “develop* lifecycle” OR “design* lifecycle” OR “construct* lifecycle” OR “implement* lifecycle” OR “develop* platform*” OR “design* platform*” OR “construct* platform*” OR “implement* platform*”))	3	3	3	3
IEEE	(“Index Terms”:IoT OR “Index Terms”:”Internet of Things”) AND (“Index Terms”:”develop* method*” OR “Index Terms”:”design* method*” OR “Index Terms”:”construct* method*” OR “Index Terms”:”implement* method” OR “Index Terms”:”develop* framework” OR “Index Terms”:”design* framework” OR “Index Terms”:”construct* framework” OR “Index Terms”:”implement* framework” OR “Index Terms”:”develop* tool*” OR “Index Terms”:”design* tool*” OR “Index Terms”:”construct* tool*” OR “Index Terms”: “implement* tool*” OR “Index Terms”:”develop* guidelines” OR “Index Terms”:”design* guidelines” OR “Index Terms”:”construct* guidelines” OR “Index Terms”:”implement* guidelines” OR “Index Terms”:”develop* lifecycle” OR “Index Terms”:”design* lifecycle” OR “Index Terms”:”construct* lifecycle” OR “Index Terms”:”implement* lifecycle” OR “Index Terms”:”develop* platform*” OR “Index Terms”:”design* platform*” OR “Index Terms”:”construct* platform*” OR “Index Terms”: “implement* platform*”)	452	429	429	40
WoS	TS=(IoT OR “Internet of Things”) AND TS=(“develop* method*” OR “design* method*” OR “construct* method*” OR “implement* method” OR “develop* framework” OR “design* framework” OR “construct* framework” OR “implement* framework” OR “develop* tool*” OR “design* tool*” OR “construct* tool*” OR “implement* tool*” OR “develop* guidelines” OR “design* guidelines” OR “construct* guidelines” OR “implement* guidelines” OR “develop* lifecycle” OR “design* lifecycle” OR “construct* lifecycle” OR “implement* lifecycle” OR “develop* platform*” OR “design* platform*” OR “construct* plat-form*” OR “implement* platform*”)	3201	2475	2344	83
Scopus	KEY ((IoT OR “Internet of Things”) AND (“develop* method*” OR “design* method*” OR “construct* method*” OR “implement* method” OR “develop* framework” OR “design* framework” OR “construct* framework” OR “implement* framework” OR “develop* tool*” OR “design* tool*” OR “construct* tool*” OR “implement* tool*” OR “develop* guidelines” OR “design* guidelines” OR “construct* guidelines” OR “implement* guidelines” OR “develop* lifecycle” OR “design* lifecycle” OR “construct* lifecycle” OR “implement* lifecycle” OR “develop* platform*” OR “design* platform*” OR “construct* platform*” OR “implement* platform*”))	647	646	636	60

**Table 2:** Methodologies focused on IoTS development, and the artifacts used and/or recommended to achieve their objectives.

Year	Reference	Requirements *	Artifacts for			Approach	Application
			Modelling Language	Analysis and Modelling	Code Generation		
2018	Lekidis et al. [168]	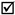	✗	DSML ^a^	C/C++	MDE, SOA ^b^	Smart Buildings
2017	MDE4IoT ^c^ [172]	✗	UML, DSML	✗	Java, C/C++	MDE	Smart Cities
2017	IOPT ^d^ [174]	✗	Petri net	IOPT networks	ANSI C	Petri Net	Smart Car
2017	Brambilla et al. [170]	~	mobile IFML ^e^	~	NS ^f^	Components and patterns	Several domains
2017	Harbouche et al. [169]	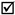	UML	AD ^g^, SD ^h^	NesC, Java	MDE	Health Care
2016	Chauhan et al. [175]	DL ^i^	Holder	AL ^j^, UIL ^k^	Node.js	MDD	Smart Home
2012	ROOD [176]	NDE ^l^	SsML ^m^	SOM ^n^	J2ME ^o^	MDA	Smart Gym
2012	ELDAMeth [171]	~	✗	ELDA ^p^, MMM ^q^	Java	Agents	Mobile Agents

^a^ Domain-Specific Modelling Language; ^b^ Service-Oriented Architecture; ^c^ MDE for IoT; ^d^ Input-Output Place-Transition; ^e^ Interaction Flow Modelling Language; ^f^ NOT specified; ^g^ Activity Diagrams; ^h^ Sequence Diagrams; ^i^ Domain Language; ^j^ Architecture Language; ^k^ User Interaction Language; ^l^ Environmental Context Model; ^m^ Smart Space Modelling Language; ^n^ Smart Object Model; ^o^ Java 2 Micro ©Edition; ^p^ ELDA (Event-driven Lightweight Distilled state charts-based Agents) [177]; ^q^ MAS (Multi-Agent System) Meta-Model. 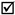 Very important; ~ Mentioned; ✗ They don’t mention them; * Collection and analysis of requirements.

**Table 3:** Methodologies specifying the stages of the life cycle.

Year	Name	Bases (Methodology/Approach)	(1)	(2)	(3)	(4)	(5)	(6)	Other
2022	RASPSS ^a^ [180]	DDD ^b^	✗	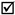	✓	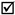	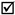	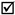	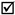
2021	Schauer and Falas [181]	AS ^c^	✗	✓	✓	**~**	**~**	✗	✗
2020	TDDM4IoTS ^d^ [37]	Agile	✓	✓	✓	✓	✓	✓	✓
2019	Pico-Valencia et al. [94]	Agile (SCRUM)	**±**	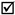	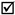	✓	✓	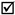	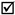
2019	Gogineni et al. [86]	V Model XT	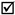	✓	✓	✓	✓	✗	✗
2018	INTER-METH [65]	Iterative waterfall	**~**	✓	✓	✓	✓	✓	✗
2018	SERVUS [102]	SOA	✗	✓	✓	✓	✓	✗	✗
2018	Sosa-Reyna et al. [123,124,125]	MDD and SOA	✗	✓	✓	✓	✓	✗	✗
2017	SEM ^e^ [182]	Metamodel	✗	✓	✓	✓	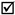	✗	✗
2017	Arrowhead [183]	SOA ^f^	✗	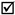	✓	✓	✓	✗	✓
2016	IDeA ^g^ [184]	MBSE ^h^, OOSEM ^i^	✗	✓	✓	**±**	**±**	**±**	**±**
2015	Patel and Cassou [78]	Concerns-Oriented	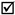	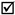	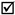	✓	✓	✓	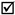
2015	Fortino et al. [185]	Metamodel	**~**	✓	✓	✓	✗	✗	✗
2013	AMG [38]	Model Transformation	✗	**~**	✓	✓	✗	✗	✗

^a^ Rehabilitation Assistive Smart Product-Service System; ^b^ Data-Driven Development; ^c^ Atomic Services; ^d^ Test-Driven Development Methodology for IoTSs; ^e^ Smart Environment Metamodel; ^f^ Service-Oriented Architecture; ^g^ IoT DevProcess and AppFramework; ^h^ Model-Based Systems Engineering; ^i^ Object-Oriented Systems Engineering Method.

**Table 4:** Main features of platforms, frameworks, and tools for the development of IoTSs.

Year	Reference Name	Approach	Artifacts	Final Product
2021	Autolink [189]	Components, Templates, MDD	Code, Device design diagram	IoT device: software and design
2020	TinyLink [190]	Components, Templates, MDD	Code, Device design diagram	IoT device: software and design
2018	PrIoT [154]	Components	PrIoT-core, PrIoT-API, Prior-Test, Prior-DB, PrIoT-UI	Conceptual framework
2018	Cai et al. [191]	MDA, patterns, and ontologies	BPMN, CD, AD	Software
2017	COMFIT [192]	MDA	Components	Code in NesC and C languages
2017	EDG [193]	Components (APIs), MDD	Code, BD ^a^	IoT device: software and design

^a^ Block Diagram.

## Data Availability

The data presented in this study are available on request from the corresponding author. The data are not publicly available due to privacy restrictions.
